# Effect of age-related extended high frequency hearing loss on the subjective impressions of dental drill noise

**DOI:** 10.1038/s41598-024-65429-y

**Published:** 2024-07-08

**Authors:** Tomomi Yamada, Sonoko Kuwano, Shigeyuki Ebisu, Mikako Hayashi

**Affiliations:** 1 Department of Restorative Dentistry and Endodontology, Osaka University Graduate School of Dentistry, Suita, Osaka 565-0871 Japan; 2 Osaka University, Suita, Osaka 565-0871 Japan

**Keywords:** Age-related hearing loss, Dental drill, Noise, Extended high frequencies, Fear, Audiometry, Psychology, Dental equipment, Dental psychology, Dental treatments, Mechanical engineering

## Abstract

Fear and anxiety among patients are sometimes evoked in dental clinics due to the sound of dental drills. This study aimed to explore the impact of age-related hearing loss in the extended high frequency (EHF) range above 8 kHz on individuals' subjective discomfort towards dental drill noise. After measuring pure-tone audiometric thresholds at both conventional and extended high frequencies, we used a psychoacoustic approach to evaluate subjective impressions of four dental drill sound stimuli, which featured varying frequency components, in 62 participants (aged 12–67 years). We found a significant decrease in hearing sensitivity within the EHF range as age increased, with notable differences in hearing thresholds at 14 kHz between teenage and older adults exceeding 65 dB. Furthermore, significant differences were observed between younger and older (above 40 years) participants in the subjective impressions of dental drill noise, emphasizing age as a critical factor in the perception of high frequency components. Consequently, age may influence the unpleasantness of dental drilling noise. Compared to older individuals, young participants may exhibit increased fear of dental procedures owing to physiological factors. These results underscore the need for age-appropriate noise control strategies in dental clinics to mitigate anxiety and improve patient comfort.

## Introduction

A variety of sounds in the dental clinic are generated from the instruments used during treatment^1^. The most notable sound in dental clinics is likely the noise produced by a dental air turbine handpiece (i.e., a dental drill)^2^, which can negatively impact patients^3^. A survey on patients’ impressions of various dental situations showed that approximately half of the respondents experienced unpleasant feelings upon hearing drilling sounds during dental treatment, comparable to fears associated with pain or dental anaesthesia injections^3^. Further studies have highlighted that individuals with high levels of dental anxiety are more likely to avoid dental procedures^3,4^ and consequently suffer from poorer oral health^5,6^. An existing study reported that over 60% of people with moderate to high dental fear avoid dental treatment, even when necessary^7^. These findings underscore the importance of creating a comfortable acoustic environment in dental clinics to enhance patient comfort and promote better oral health outcomes.

In general, noise control aims to reduce noise levels. The equivalent continuous A-weighted sound pressure level (*L*_Aeq,_ dB) is an energy-based metric widely used to evaluate temporally varying sounds and environmental noise. Numerous reports have demonstrated a significant correlation between subjective evaluations, such as perceiving an 80-dB sound subway as loud and a 40-dB library as quiet, and *L*_Aeq_ values^8–12^. *L*_Aeq_ correlates well with human physiological and psychological reactions to noise^8^. Adopted as a standard measure of environmental noise by the International Organization for Standardization (ISO) in 1996^9^, *L*_Aeq_ has also been integrated into Japanese environmental standards^10^. In addition to reducing noise levels, efforts are being made to improve sound quality. Manufacturers and researchers are actively working to improve and design preferred, quieter, more pleasing and easier-to-hear sounds^13–16^. The Noise Criterion value in the U.S. and the Noise Rating curve in Europe are established standards that define acceptable sound pressure levels for frequencies up to 8 kHz^17,18^. These criteria are crucial for ensuring suitable indoor environments for air-conditioning equipment, preserving hearing, facilitating speech communication, and reducing annoyance. They primarily target frequency bands below 8 kHz, which include the range most sensitive to the human ear^19^. However, the sound emitted by various dental drills under drilling condition includes prominent frequency components above 8 kHz^20^, presenting unique challenges that current noise control measures may not adequately address.

Humans can hear frequencies ranging from 20 Hz to 20,000 Hz. Frequencies beyond this audible range are considered to be in the ultrasonic region^21^. In speech and phonetic literature, ‘high frequency’ often refers to frequencies between 2 and 5 kHz and occasionally up to 6 to 8 kHz^19,22^. While standard audiometry test frequencies range from 0.125 kHz to 8 kHz, the extended high frequency (EHF) range includes higher frequencies above 8 kHz^23^. The ‘high frequency range’ below 8 kHz plays a crucial role in human hearing, particularly in understanding language, distinguishing sounds, and discerning music details. Conversely, it is widely assumed that the EHF range plays little or no role in speech perception owing to evidence that energy of approximately 7 kHz or less is sufficient to reproduce clear speech for transmission through communication systems^24^. EHF audiometry is not commonly included in standard auditory examinations and requires specialized equipment and techniques^25,26^. The EHF range, spanning from 8 to 20 kHz, represents a region of the human hearing spectrum that is generally ignored by both clinicians and researchers^24^ and, as of 2022, the EHF range has rarely been considered in audiometric testing in clinical or research settings^27^.

The ISO provides information indicating that the sensitivity of human hearing decreases more rapidly with age for high frequency sounds than for low-frequency sounds^28^. As individuals age, their ability to hear high frequencies between 2 and 5 kHz declines, which can make it difficult to understand speech^24^. This decrease in hearing is known as presbycusis or age-related hearing loss, which primarily affects the perception of ‘high frequency’ sounds. A previous study reported that approximately half of the individuals with dental anxiety experienced dental fear in their childhood^29^. Research investigating the causes of dental fear and anxiety^3–7,29–31^ has identified exogenous (direct learning from traumatic experiences, vicarious learning through family and the media) and endogenous factors (inherited and personality traits)^29–31^. However, the effect of physiological factors such as EHF hearing on subjective impressions of dental drill noise has not been previously investigated. We hypothesized that young patients would have sufficient hearing to perceive the frequency components in the EHF of dental drill sounds. As a result, they may experience dental drill sounds as louder and more unpleasant, potentially leading them to avoid dental care.

This study aimed to elucidate how age-related hearing loss in frequencies above 8 kHz influences individuals' subjective impressions of dental drill noise. We suggest that this understanding may lead to more effective noise mitigation strategies in dental care environments. This exploration into the intersection of auditory health and patient comfort in dental care marks a significant step towards optimizing the auditory experience for patients of all ages. In this study, we conducted EHF audiometry to understand the physiological differences in hearing among participants. We then conducted psychoacoustic experiments for dental drill sounds with different frequency components using the semantic differential method^11^. Participants were asked to rate their subjective impressions of the sound, such as loudness and unpleasantness. This psychoacoustic approach is often used to identify the relationships between subjective impression and the objective physical properties of the sound, such as sound pressure levels^11–16,32,33^. Only a few studies have investigated the impact of EHF components in noise on perceptions shaped by age-related hearing ability, making this investigation a novel contribution to the fields of audiology, psychology, and acoustical physics^24^. Furthermore, the findings of this study offer significant insights for both patients who fear dental treatment and the dentists who treat them. These results are expected to clarify the differences in perception levels of dental drill noise across different age groups.

## Methods

This study was approved by the ethics committee of the Osaka University Graduate School of Dentistry and conformed to the tenets of the Declaration of Helsinki. Written informed consent was obtained from all participants or their guardians (for participants aged under 18 years) before the commencement of the study.

### Participants

Sixty-two participants (male, 26; female, 36) aged 12–67 years participated in this study. A broad age range was targeted to encompass the developmental spectrum of hearing. However, a lower age limit of 12 years was established to ensure that participants could accurately report their audiometry and psychoacoustic experiments. Participants completed a preliminary questionnaire in which they were asked to report any history or current occurrence of hearing difficulties, such as sudden sensorineural hearing loss or chronic ear infections. There were no participants who met the exclusion criteria of having a positive history and currently hearing difficulties. All participants were Japanese. Participants were divided into three groups based on age: teenagers (under 18 years), young adults (between 18 and 39 years), and older adults (40 years and above). The teenage group consisted of 25 participants aged 12–17 years (average age: 14.5 years). The young adult group comprised 23 participants aged 21–39 years (average age: 34.0 years), including 21 in their 20s and three in their 30s. The older adult group included 14 participants aged 42–67 years (average age: 54.7 years), with four in their 40s, seven in their 50s, and three in their 60s. Hearing loss varies among individuals, but it tends to become more prevalent with age. Numerous studies have reported the onset of physiological age-related hearing loss around the age of 40 or 50 years in conventional audiometry^28,34–36^. This evidence guided our decision to define participants aged 40 years and older as 'older adults'. The decision to classify participants as teenagers (17 years and below) was driven by the need to gather more data on high frequency hearing levels in individuals under 18 years. Due to the scarcity of such information, this study emphasizes the need to focus on younger age groups.

### Audiometric test

To ascertain participant minimum hearing thresholds, a pure-tone audiometer (UNITY 2; Siemens Audiologische Technik GmbH, Erlangen, Germany) equipped with earphones (HDA 200, Sennheiser Electronic GmbH & Co.KG, Wedemark, Germany) was utilized. The calibration of the audiometer to the reference equivalent threshold sound pressure levels (RETSPLs) for pure tones up to 16 kHz, as dictated by the ISO 389-5^25^ and 389-8^26^ standards, ensured measurement precision and adherence to international hearing assessment protocols. The unit of the audiometry was the hearing level (HL). The 0 dB HLs of each pure tone are based on RETSPLs, which are defined relative to the sound pressure level (dB SPL) corresponding to the mean threshold hearing level for 18-year-olds. The minimum level of the test tones at all frequencies was set to − 10 dB HL by the audiometer. The maximum HLs at 14 and 16 kHz were limited to 75 dB HL and 55 dB HL by the audiometer, respectively, corresponding to approximately 110 dB SPL. For teenage participants, special upper limits of HLs at 14 and 16 kHz were set to 35 and 25 dB HL, respectively, corresponding to approximately 80 dB SPL. This precaution was taken because of the unclear impact of high SPL exposure in EHF on the developing auditory systems of younger individuals. Therefore, we limited the maximum exposure for teenage participants at our discretion. Audiometric testing was initiated with the conventional audiometric frequencies (1, 2, 4, and 8 kHz), moving onto the extended high audiometric frequencies (10, 12.5, 14, and 16 kHz). Each ear was assessed separately, with the starting ear (left or right) chosen randomly for each participant. Pure-tone audiometry was conducted by a speech therapist using the ascending method, which involves gradually increasing the sound level until the participant indicates perception^23^. A hearing threshold imbalance greater than 30 dB between both ears at frequencies below 8 kHz was considered an exclusion criterion because a difference of this magnitude is clearly unilateral hearing loss rather than a physiological phenomenon^37^.

### Psychoacoustic evaluation experiment

We conducted a psychoacoustic evaluation of dental drill sounds using the semantic differential method^11^. This methodological choice was predicated on its ability to transform subjective auditory experiences into quantifiable data through a structured approach. Each participant, situated alone in an acoustically isolated room, was exposed to the four dental drill sound stimuli. Participants rated their impression of the stimuli using a semantic differential scale comprising seven categories of 15 pairs of opposing adjectives, such as ‘loud-soft’. These adjective pairs, selected based on previously reported studies^20^, aimed to comprehensively quantify the range of auditory impressions elicited by the drill sounds. Prior to the start of the experiment, participants were informed that they would hear the sound of a dental drill during the experiment and received brief training with one sound stimulus that was not used in the experiment. The sound stimuli were reproduced through a high-fidelity audio system with a personal computer and presented to the participants through an audio interface (UA-4FX, Edirol, Roland Corp. Shizuoka, Japan), an amplifier (SRM-313, STAX, Saitama, Japan), and headphones (SR-303, STAX). Each participant completed two sessions, wherein the sequence of stimuli presentation was varied to mitigate order effects and enhance the reliability of the findings.

To measure the psychoacoustic impact of dental drill noises on individuals, it was necessary to prepare recorded sounds under conditions simulating the clinical use of dental drills. We previously analysed the acoustic characteristics of various dental drill sounds under different conditions, including numerous types of dental drills^20^. Artificial teeth (A20-500^38^, Nissin Dental Products Inc., Kyoto, Japan), designed with a two-layer structure that mimics enamel and dentin and used in dental education practice, were drilled to a standardized depth using each dental drill attached with a new tapered shoulder-shaped FG diamond bur (Smooth cut B1, GC dental products corp., Aichi, Japan) by an experienced dentist, thereby standardizing the drilling conditions across different dental drills. The data were recorded in a controlled environment at Osaka University Dental Hospital where ambient noise levels were minimized during non-operational hours. The dental drill was operated at manufacturer-recommended air pressure settings and utilized a water/air spray to cool the friction heat, closely simulating real-life dental procedures. To capture the sound, a ¼-inch microphone (UC-29; Rion Co., Ltd., Tokyo, Japan) was strategically placed 30 cm from the drill head. This positioning was selected as the optimal distance to minimize water interference with the microphone while still accurately recording the drill's sound. Accompanying the microphone, a precision sound level meter (NA-40; Rion Co., Ltd., Tokyo, Japan) and a digital recorder (Edirol R4-Pro; Roland Corp., Shizuoka, Japan), with a 24-bit resolution and a sample rate of 96 kHz, were employed to ensure high quality of the recordings. The use of teeth for dental training ensures not only reproducibility but also allows for condition-specific or drill-specific comparisons of dental drill noise. This approach revealed that the sound emitted from various dental drills during drilling includes an abundant EHF component, which is rarely present during idling conditions. This observation led to the formulation of the hypothesis for this study.

**Figure 1:**
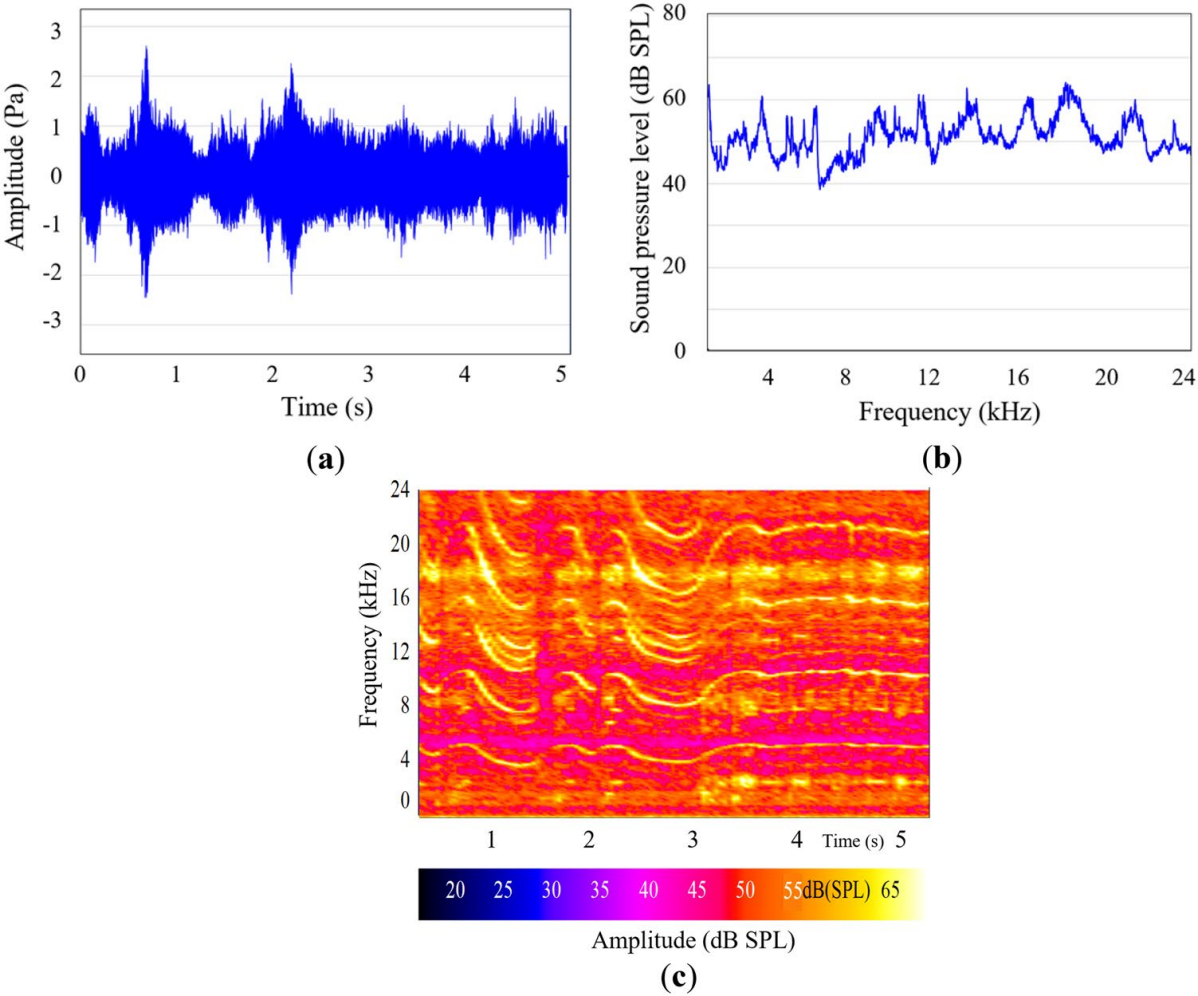
Acoustic properties of Stimulus A. **(a)** waveform (level vs. time); **(b)** fast Fourier transform (FFT) analysis results (Average of sound for 5 s); **(c)** Spectrogram (FFT vs. time). Frequency (Y-axis), time (X-axis), and amplitude (brightness of colour).

Four distinct sound stimuli (Stimuli A-D) of a dental drill (Super Torque, Kaltenbach & Voigt GmbH, Lörrach, Germany) were prepared for the psychoacoustical experiment, each with a duration of 5 s. We conducted spectrum and fast Fourier transform (FFT) analyses to investigate acoustical characteristics using ArtimiS SUITE software developed by Head acoustics (Aachen, Germany). We applied a Hanning window and computed FFT with a spectrum size of 4,096 points. Figure 1a–c shows the acoustical characteristics of Stimulus A used in a psychoacoustic experiment. Stimulus A encompassed a frequency range of up to 24 kHz, reflecting the full spectrum of the drill noise. Stimulus B, C, and D isolated specific frequency ranges for targeted analysis. Stimulus B was edited to include only the low-frequency components of Stimulus A, with frequencies limited to 8 kHz, to align with conventional acoustic analysis. Similarly, Stimulus C included low-frequency components up to 15 kHz, while Stimulus D comprised high frequency components above 15 kHz. This allowed us to examine the specific impact of these divided frequency ranges on participant perception. These stimuli were designed to explore the auditory system's response to a wide range of frequencies. The stimuli, designated as Stimuli A-D, were differentiated by their frequency components to investigate the psychoacoustic effects across a spectrum extending from conventional auditory ranges to ultrasonic frequencies. In addition, to assess the reliability of *L*_Aeq_ for subjective ‘loud-soft’ ratings in individuals with age-related hearing loss, we also investigated the relationship between *L*_Aeq_ and the rating for dental drill noise with high frequency components above 8 kHz. We calculated the *L*_Aeq_ values for each stimulus as an acoustic physical value using a sound level meter (LA1250; Ono Sokki, Yokohama, Japan).

### Statistical analyses

We analysed the quantified subjective and objective data in our study. All statistical analyses were performed using SPSS statistical software (Advanced Analytics Inc., Japan), and p values of < 0.05 were considered statistically significant.

## Results

### Data used for analysis

No participants were excluded from the study owing to unilateral hearing loss, as determined by an audiometric test. Before conducting the analyses, we examined the correlation between the results of the two psychological evaluation trials to validate the answers of each participant. A significant correlation was found among the 62 participants in the psychological evaluation (Spearman’s rank correlation, correlation coefficient r = 0.87). This result indicates that the assessments of all participants were reliable. Hence, the data of all participants were used in the analyses described below.

### Age-related hearing loss

**Figure 2:**
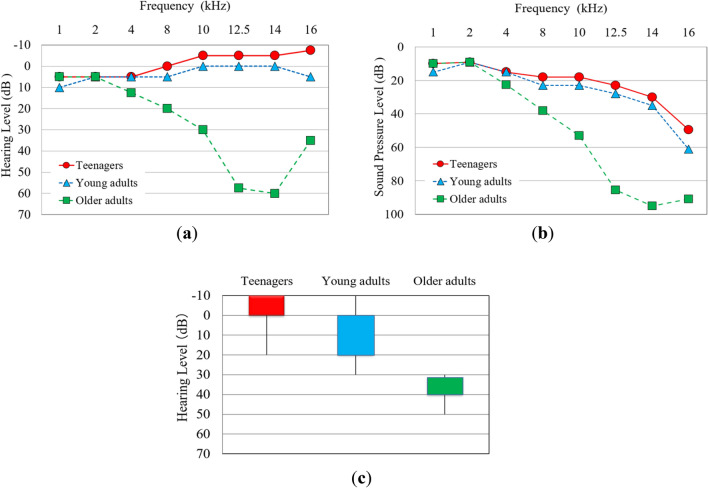
Distribution of pure-tone thresholds. (**a**,**b**) Median of the audiometry pure-tone minimum hearing thresholds of the right ear in conventional and EHF audiometry (**a** Hearing level; **b** Sound pressure level). (**c**) Interquartile ranges of audiometry thresholds of the right ear are at 16 kHz. Each box shows the ranges from the upper to lower quartile, while the vertical line represents the upper and lower extremes.

Our results highlighted age-related differences in hearing sensitivity, particularly in the EHF range above 8 kHz. Figure 2a,b show the median of the pure-tone minimum hearing thresholds for the right ear in the three age groups, with audiometry units represented in HL in Fig. 2a and converted to sound pressure level (SPL) in Fig. 2b. Figure 2a shows improved hearing levels at 16 kHz compared to those of 14 kHz in older adults and teenagers. However, there were no real improvements; these apparent improvements are merely an effect of the HL reference values, which represent the relative value of 0 dBHL. Figure 2b helps understand that perceiving pure test tone of 16 kHz required a large sound pressure level. A higher SPL was required to perceive the higher frequency of the pure test tone in the EHF across all age groups. The Kruskal–Wallis test indicated significant differences in hearing thresholds among the three age groups, with older adults showing a physiological decline in hearing sensitivity at frequencies above 8 kHz. All the participants in the three groups could perceive the pure test tones from 1 kHz up to 14 kHz. Comparisons between teenagers and older adults revealed considerable disparities in median hearing thresholds in EHF, with differences reaching 20 dB at 8 kHz, 35 dB at 10 kHz, 62.5 dB at 12.5 kHz, and 65 dB at 14 kHz. The disparity in hearing thresholds for EHF due to aging can be so significant that it may lead to a complete inability to hear high-frequency sounds, illustrating the profound impact of aging on auditory perception. All individuals in the young adult group perceived the pure test tone at 16 kHz, although there were considerable individual differences in their perception. In the teenage group, one left ear and two right ears were unable to perceive the special limited pure test tone of 25 dB HL at 16 kHz. In the older adult group, one right ear and two left ears of participants aged 60 and over could not perceive the maximum pure test tone of 75 dBHL at 16 kHz, indicating a dropout. Figure 2c shows the interquartile range of the hearing threshold levels at 16 kHz for the right ear among participants who detected a pure test tone at 16 kHz. Each box in Fig. 2c represents the range from the upper to the lower quartile, with vertical lines indicating the upper and lower limits. The results for the three age groups are presented. All older adults required higher sound pressure levels, above 30 dBHL, to perceive the sound. Figure 2c shows that the range of the distribution was 30 dB for teenagers and 40 dB for young adults. Eleven teenagers were able to hear the minimum pure test tone of − 10 dB HL at 16 kHz. One participant in their twenties and one participant in their thirties were able to hear the minimum pure test tone of − 10 dB HL at 16 kHz. These findings reveal significant large individual differences in EHF hearing ability, even among participants in the same age group.

### Effect of hearing thresholds on the perception of sound stimuli

We were able to collect data for three stimuli: Stimulus A (with high frequencies above 8 kHz), Stimulus B (with frequencies up to 8 kHz), and Stimulus C (with frequencies up to 15 kHz) across the three age groups in the psychological evaluation experiment. However, for Stimulus D, which consisted only of higher frequency components above 15 kHz, data could not be obtained from the older adult group because all 14 participants were unable to detect Stimulus D. Furthermore, complete data for Stimulus D could not be obtained for five of the 25 teenagers and seven of the 23 young adults, as these participants could not perceive the stimulus.

Referring to the audiology test results, two of the five teenagers who could not perceive Stimulus D were outscaled at the limited pure test tone of 25 dB HL. For the remaining three teenagers, the average threshold level at 16 kHz for both ears was 15 dB HL. In the young adult group, the average threshold level for both ears of the six participants who could not detect Stimulus D was 20 dB HL at 16 kHz, whereas for those who could detect it, the average was 5 dB HL. The entire older adult cohort failed to perceive Stimulus D, with their minimum audible thresholds exceeding 30 dB HL. Consequently, it was inferred that individuals with a minimum hearing level of approximately 15 dB HL or greater at 16 kHz were physiologically unable to perceive the sound in Stimulus D.

### Effect of age-related hearing loss on efficacy of *L*_Aeq_

**Figure 3:**
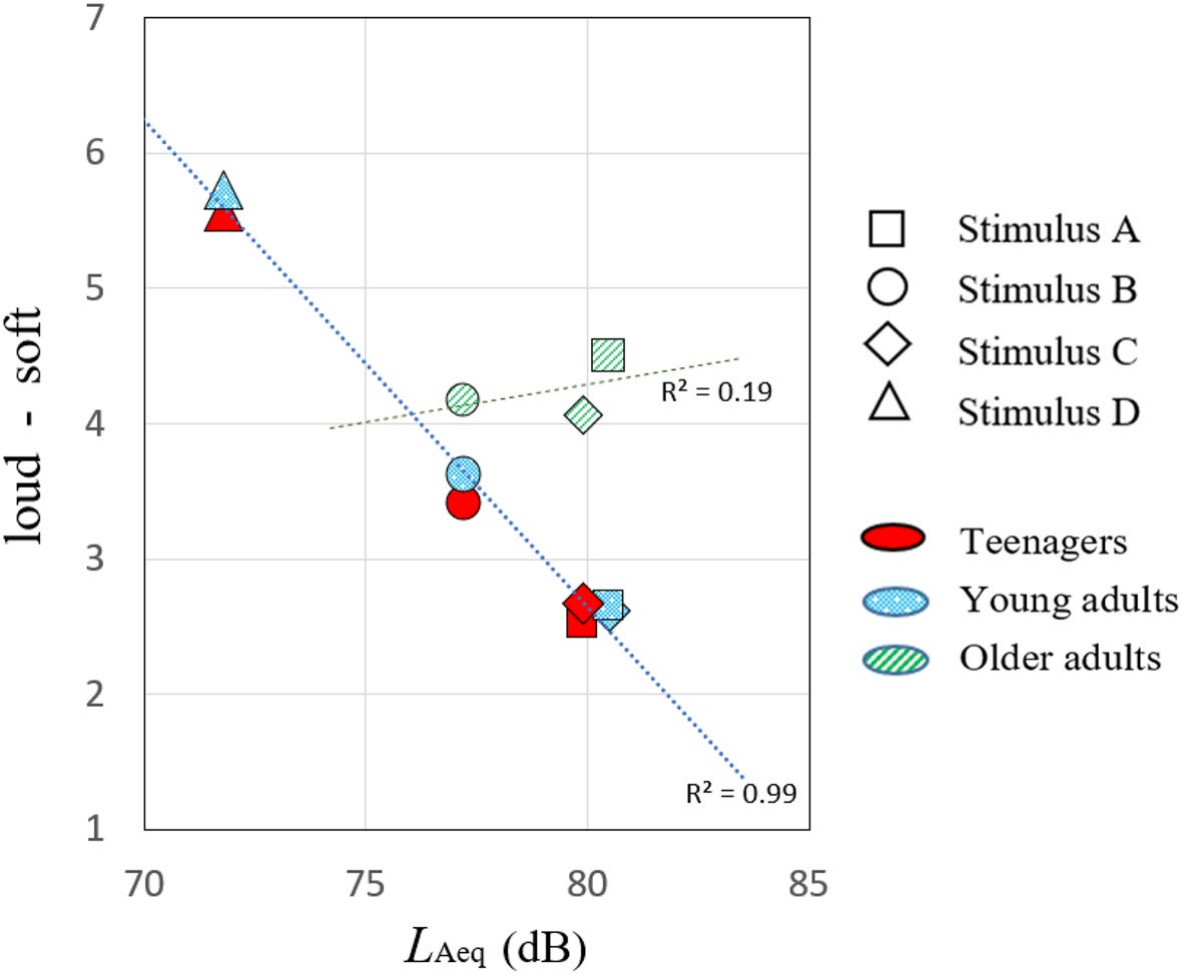
Relationships between the *L*_Aeq_ values and the score of the subjective ‘loud-soft’ among the three age-groups. Square, round, diamond, and triangle marks were the results of Stimulus A, Stimulus B, Stimulus C, and Stimulus D, respectively. Red, blue, and green colours indicate the results of the teenagers, young adults, and older adults, respectively.

The study explored the correlation between semantic differential judgments of ‘loud-soft’ on a seven-point scale and the physical values of *L*_Aeq_ across age groups, aiming to evaluate the efficacy of *L*_Aeq_ as a dependable metric. The *L*_Aeq_ values of stimulus A and C were higher than those of stimulus B, and stimulus D had a significantly smaller *L*_Aeq_ value. Figure 3 shows the relationships between the *L*_Aeq_ values of the stimuli and subjective ‘loud-soft’ impressions, revealing a strong correlation between *L*_Aeq_ values and ‘loud-soft’ scores among both teenagers and young adults (R^2^ = 0.99). Conversely, this correlation was absent among older adults. These outcomes suggest that *L*_Aeq_ is a valuable metric for gauging loudness for both teenagers and young adults who retain high frequency hearing capabilities. In contrast, for older adults, loudness judgments based on *L*_Aeq_ for sounds of dental drills, enriched with high frequency components, become unreliable. This discrepancy highlights the variability in auditory processing and perception mechanisms across different age groups, underlining the importance of considering these differences in noise control measures.

### Subjective evaluations of dental drill noise among the three age groups

**Figure 4:**
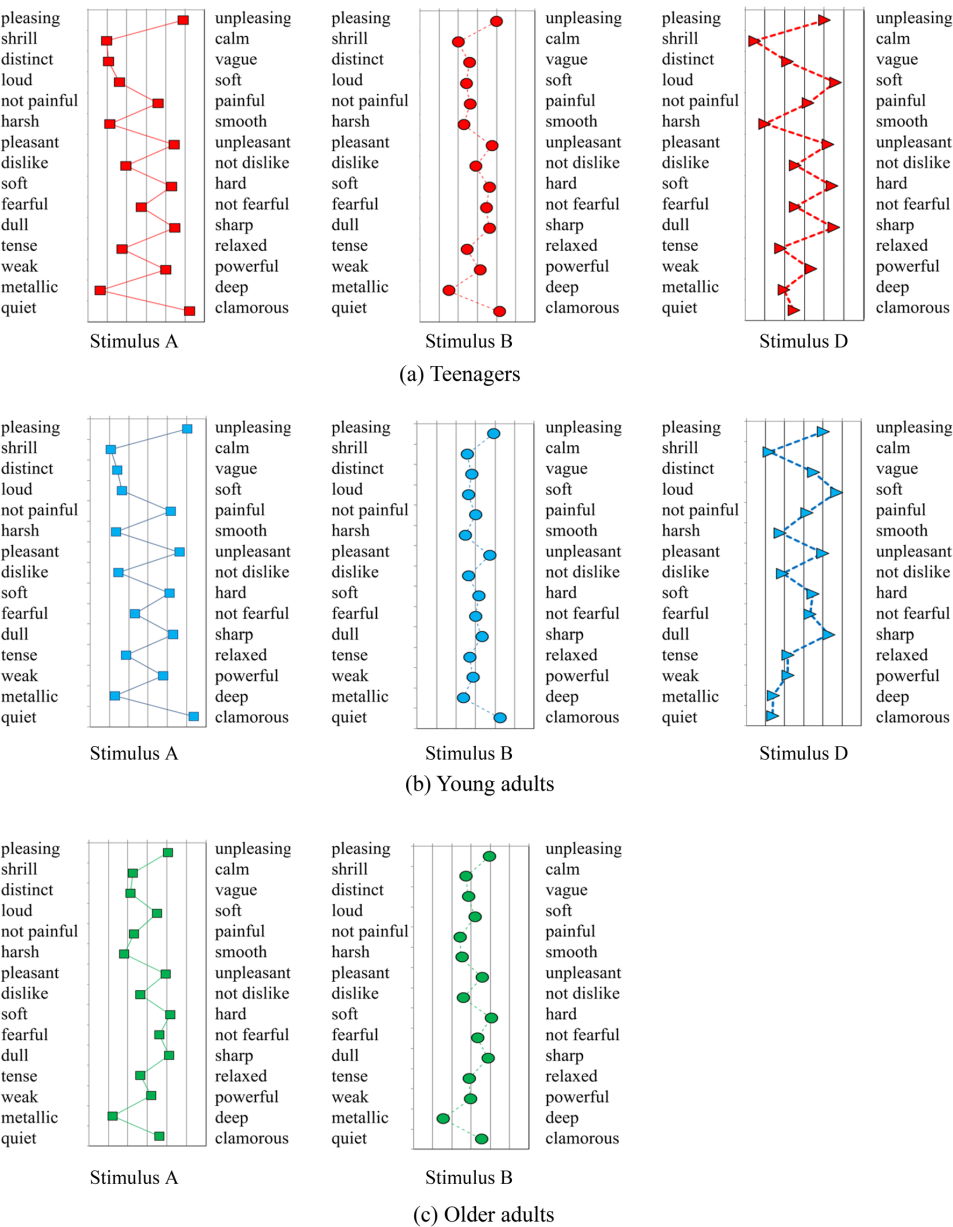
Average rating of 15 adjective pairs for subjective impressions in the three groups. (**a**) Teenagers, (**b**) Young adults, and (**c**) Older adults.

Various impressions were assessed in addition to ‘loud-soft’. Figure 4 shows the average subjective impressions of the stimuli across the three age groups. The rating of stimulus B was similar across the age groups. Teenagers and young adults showed significant differences in their evaluations of Stimuli A (with high frequencies above 8 kHz) and B (without high frequencies above 8 kHz), as confirmed by Levene's test for homogeneity of variance and the Mann–Whitney *U* test (two-tailed p-value < 0.05). Conversely, older adults found no significant distinction between Stimuli A and B, except for the descriptors ‘harsh-smooth’ and ‘distinct-vague’. Responses to Stimulus C (frequencies up to 15 kHz) were similar to those to Stimulus A within each age group. These results suggest that perceptual abilities up to 15 kHz in EHF affect considerable to various subjective impressions. Teenagers and young adult participants who could perceive Stimulus D, characterized by a higher frequency of components above 15 kHz, assessed this as much ‘softer’ (vs. loud) and ‘quieter’ (vs. clamorous) than Stimulus A. Notable, stimulus D, as well as Stimulus A, was rated as ‘shrill’, ‘harsh’, ‘hard’, ‘unpleasant’, ‘dislike’, ‘sharp’, and ‘tense’. These findings suggest that for participants with good hearing ability in EHF, high frequency components closer to the ultrasonic range also contributed to sound impressions regarding sharpness and unpleasantness.

### Factors composing the subjective impression of dental drilling noise

Factor analysis of the data for dental drilling noise was conducted using the Varimax rotation method. Factor analysis in psychoacoustics is a statistical method that extracts common factors or structures from a subjective assessment of sound. Through this analysis, we gained insight into the factors of sound that contribute to people feeling comfortable or uncomfortable. These findings contribute to designing appropriate sound quality assessment in the development of acoustic products or noise control planning. Two factors were extracted from the analysis of all the data, as shown in Table 1. For example, the adjective pair ‘shrill-calm’ had a high correlation with Factor 1 (0.959) and almost no relation to Factor 2 (0.016). This indicates that Factor 1 was primarily associated with adjectives such as ‘shrill-calm’, ‘harsh-smooth’, ‘dull-sharp’, and ‘tense-relaxed’, characterizing the dimension of sound 'sharpness'. In contrast, Factor 2 included adjective pairs such as ‘quiet-clamorous’, ‘loud-soft’, and ‘weak-powerful’ with a high correlation, which represent the ‘loudness’ dimension. Factor 2 is associated with the perception of loudness versus softness, while Factor 1 relates to the perception of calmness versus sharpness. Adjectives such as ‘unpleasant’, ‘painful’, ‘dislike’, ‘fearful’, and ‘pleasing’ were linked to both factors. These results suggest that the major subjective impression for dental drilling noise comprised both sharpness (Factor 1) and loudness (Factor 2). The cumulative variance accounted for by these factors was 81.2%, with Factor 1 contributing 47.7% and Factor 2 contributing 33.5%. Thus, it shows that perceptions of unpleasantness for dental drilling noise are influenced not only by the loudness but also by the sharpness of the sound.

**Table 1:** Correlation coefficient between each adjective pair and the two factors.

	Factor 1	Factor 2
shrill-calm	0.959	− 0.016
harsh-smooth	0.938	− 0.195
dull-sharp	− 0.925	0.045
tense-relaxed	0.925	− 0.24
pleasant-unpleasant	− 0.862	0.442
not painful-painful	− 0.753	0.468
dislike-like	0.747	− 0.352
fearful-not fearful	0.715	− 0.388
soft-hard	− 0.556	0.364
metallic-deep	0.433	− 0.536
pleasing-unpleasing	− 0.618	0.736
distinct-vague	0.472	− 0.817
weak-powerful	− 0.391	0.843
loud-soft	0.019	− 0.973
quiet-clamorous	− 0.028	0.982

### Differences in subjective impressions among age groups as revealed by the factor score plot

**Figure 5:**
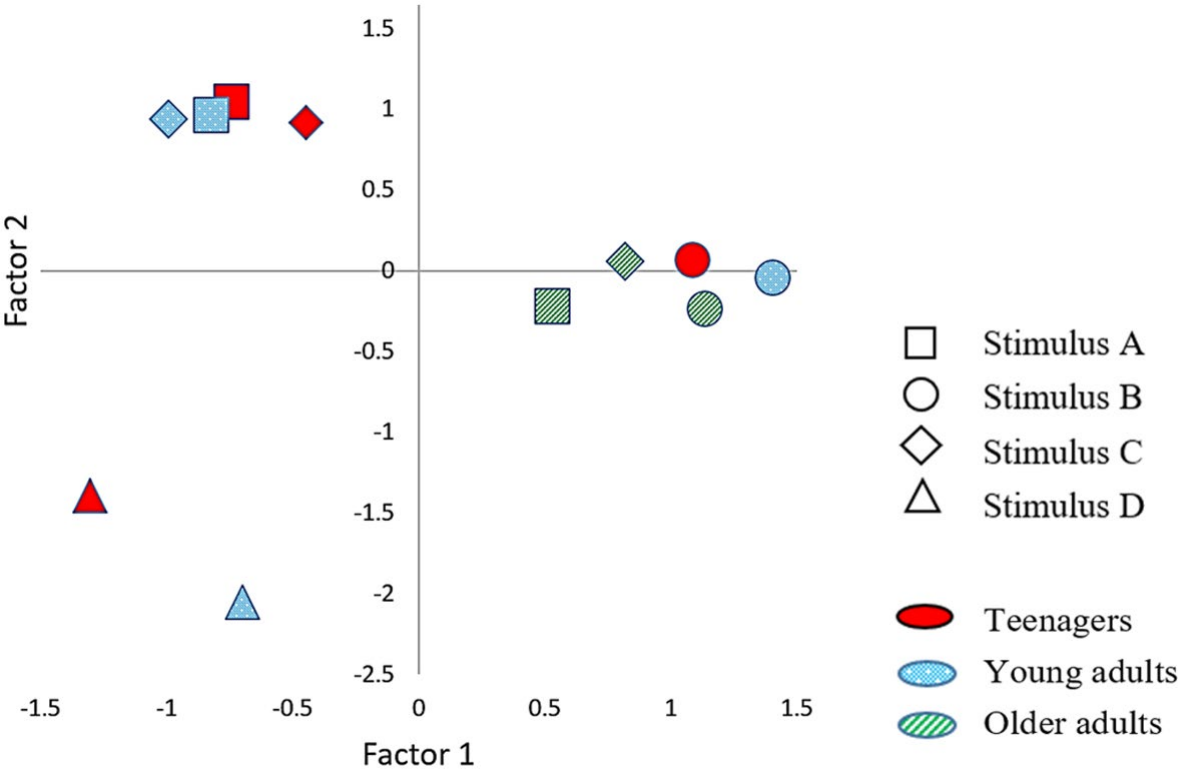
Plot of each stimulus for the three age-groups based on the loading scores of the two factors measured using factor analysis. The Y-axis represents Factor 2, which is associated with the perception of loudness versus softness, while the X-axis represents Factor 1, which relates to the perception of calmness versus sharpness.

We utilized factor scores, determined through factor analysis, to explore how subjective impressions of each sound stimulus vary across different age groups. These scores represent the strength of the influence of each factor. Figure 5 illustrates the plot of the impressions of stimuli A-D based on the calculated scores of the two factors. The Y-axis represents Factor 2, which is associated with the perception of loudness versus softness, while the X-axis represents Factor 1, which relates to the perception of calmness versus sharpness. The results for Stimulus B (indicated by a circle) for the three age groups were closely clustered together near the positive X-axis for Factor 1, indicating that teenagers, young adults, and older adults shared a similar subjective perception of dental drill noise below 8 kHz. Among the older adults, although there was a slight difference in Factor 1, Stimuli A, B, and C were plotted near the positive X-axis, indicating that the three sounds resulted in a similar impression. Meanwhile, among teenagers and young adults, Stimuli A and C were plotted in the second quadrant, and stimulus D was in the third quadrant, as shown in Fig. 5. This indicates that teenagers and young adults had a completely different impression from Stimulus B. Conversely, older adults, assessed the sound of Stimuli A, B, and C similarly.

## Discussion

Recognizing the necessity of higher sound pressure levels to perceive EHF frequencies is essential. This study highlights the critical role of sound pressure levels in the perception of EHF and suggests implications for auditory research and practical applications. Combining audiometry and psychoacoustic evaluation tests, we concluded that the significant frequency components in the rich EHF of dental drill noise were perceptible to many teenagers and young adults. Age-related hearing loss may occur because of the heightened susceptibility of inner ear hair cells to aging, which particularly impacts the perception of high frequency sounds^24,27^. Overseas studies indicate that high frequency hearing loss, particularly at 8 kHz, commences in individuals over 40 years of age^28,37^. In contrast, Japanese reports suggest that hearing decline typically begins over the age of 50 years^35,36^. Our results in the EHF range, which showed a significant decline in individuals over the age of 40 years (Fig. 2a), support previous findings that ‘age-related decline in the EHF region first becomes “clinically significant” in 36–45-year-olds’ and that hearing in the EHF range is highly age-dependent^24^. As a result, individuals over the age of 40 years should be recognized as ‘older’ in the auditory assessment of the EHF range. Some reports indicate that EHF loss begins from early adulthood^39^. However, our findings suggest that some teenagers may experience hearing loss even earlier, as shown in Fig. 2c.

The logarithmic nature of decibels in audiology illustrates that even minor numerical increases can significantly alter perceived loudness. For example, an increase of 10 dB is perceived as approximately a two-fold increase in loudness, while increases of 20 dB and 30 dB are perceived as a four- to eight-fold increase in loudness, respectively^12^. This principle is crucial for understanding the discussion on age-related hearing loss and its impact on sound perception, particularly in EHF. Frequency components within EHF that are audible to younger individuals may be barely audible or completely inaudible to older adults owing to age-related hearing loss, as exemplified by a 65 dB disparity in hearing thresholds at 12.5 kHz and 14 kHz (Fig. 2). Even if older adults can hear frequency components of EHF, the perceived loudness will be different from that of teenagers, depending on the difference between the noise level and their minimum threshold level. The findings indicate that dental drilling noise is perceived as considerably louder and sharper by younger individuals than older ones, highlighting the need to understand patient discomfort during dental procedures. Dentists and guardians may attribute a child’s reluctance to undergo dental treatment to personality or mental immaturity when, in fact, the behaviour may be caused by physiological characteristics such as superior high frequency hearing ability.

Our results indicate the necessity for age-specific psychological countermeasures to alleviate dental fear and anxiety in dental offices. This critical finding suggests that age-related hearing loss in EHF significantly affects not only the perceived quantity but also the quality of sounds, providing valuable insights for noise control and environmental design considerations. Furthermore, the reliability of *L*_Aeq_ for assessing loudness aids in developing effective countermeasures. Using headphones or earplugs might mitigate the impact of air-conducted sound to some extent; however, addressing the transmission through teeth (teeth-conducted sound) during dental procedures remains a challenge. This aspect of noise management and hearing protection strategies in dentistry requires further research. We will continue to address this challenge as managing dental noise is essential for ensuring patient comfort during treatment. This, in turn, can contribute to reducing healthcare costs and improving oral health.

Additionally, the scope of EHF research expands into various fields beyond dentistry, such as environmental studies and behavioural science. Rich frequency components in EHF and ultrasonic ranges are contained in squeaking noise of high-speed trains and devices to repel cats and mice in public places^21^. Our findings could elucidate why younger individuals find certain sounds particularly unpleasant, contributing to research on how high frequency hearing loss affects daily life experiences, thereby leading to more inclusive auditory health strategies.

This study has some limitations. First, owing to constraints with the audiometry equipment, audiometry could not be conducted below – 10 dB HL across all test frequencies up to 16 kHz. Additionally, we did not perform audiometry above the restricted threshold of 16 kHz. Consequently, the scope of individual variability in hearing abilities among teenagers and young adults might be broader, with some auditory abilities potentially being much better than our results suggest. Second, no real human teeth, including extracted teeth, were used in this study. We consider that the use of artificial teeth does not significantly compromise the validity of our findings regarding the perception of dental drill noise. Extracted teeth are often structurally compromised and may exhibit dry conditions. This approach not only maintains the consistency of the drilling conditions but also closely mirrors a real clinical scenario.

## Conclusions

This study explored the impact of age-related hearing loss in the EHF range above 8 kHz on individuals' subjective experiences of dental drill noise. Our results support the hypothesis that age considerably influences the perception of high frequency components in dental drill sounds. Particularly, younger individuals under the age of 40 years exhibited heightened sensitivity to EHF components in dental drilling noise that were less perceptible or entirely inaudible to older adults. Dental sound under drilling conditions was perceived as louder, harsher, and more unpleasant by younger individuals, leading to more negative subjective impressions of dental drill noise. These findings clarify that age significantly impacts the perception of high frequency sound components, making dental drill noises more unpleasant for younger populations, emphasizing the role of auditory sensitivities above 8 kHz in shaping subjective impressions of sound. The present findings underscore the importance of considering age-related auditory when designing noise control measures in dental clinics. These results suggest that adjusting sound quantity and quality to accommodate the auditory sensitivities of different age groups could create a more comfortable acoustic environment and reduce anxiety. Implementing age-appropriate noise strategies in dental practices can enhance patient comfort, reduce dental anxiety, and encourage regular dental visits, ultimately leading to better oral health outcomes.

## Data Availability

The datasets generated and analysed during the current study are included in the published article. Some datasets are not publicly available for privacy reasons but are available from the corresponding author upon reasonable request.
